# PRODIGY score predicts respiratory depression in the post-anesthesia care unit: A post-hoc analysis

**DOI:** 10.17305/bb.2024.10585

**Published:** 2024-12-01

**Authors:** Anuradha Kanaparthi, Frances Chung, Peter R Lichtenthal, Juraj Sprung, Toby N Weingarten

**Affiliations:** 1 Department of Anesthesia and Perioperative Medicine, Mayo Clinic, Rochester, MN, USA; 2 Department of Anesthesia and Pain Management, University Heath Network, University of Toronto, Toronto, ON, Canada; 3 Department of Anesthesiology, University of Arizona Medical Center, Tucson, AZ, USA

**Keywords:** Postanesthesia care unit, PRODIGY score, postoperative respiratory depression, respiratory depressive episodes, general care ward

## Abstract

Surgical patients who experience respiratory depressive episodes (RDEs) during their post-anesthesia care unit (PACU) admission are at a higher risk of developing subsequent respiratory complications in general care wards. A risk assessment tool for PACU RDEs has not been previously assessed. The PRediction of Opioid-induced respiratory Depression In patients monitored by capnoGraphY (PRODIGY) score is an assessment tool that uses baseline patient variables to categorize patients into low-, intermediate-, or high-risk groups for RDEs in general care wards. This study assessed whether PRODIGY groups are associated with PACU RDEs. This analysis utilized data from a previous observational trial of PACU RDEs detected by capnography. PRODIGY scores were retrospectively calculated, and the number and duration of respiratory alerts were compared among PRODIGY groups. Twenty-six (29.9%) patients were classified as low risk, 29 (33.3%) as intermediate risk, and 32 (36.8%) as high risk. A total of 3580 alerts were recorded in the PACU, 47% of which were apnea episodes lasting ≥ 10 s. The total number and duration of alerts were highest in high-risk group patients (median 56 [IQR 12–87] alerts per patient vs 22 [9–37] in low-risk and 26 [13–42] in intermediate-risk patients, *P* ═ 0.035; 303 [123–885] s vs 177 [30–779] in low-risk and 301 [168–703] in intermediate-risk patients, *P* ═ 0.042). Poisson regression analysis indicated that the rate of RDEs in the high PRODIGY risk group was higher than in the intermediate (rate ratio estimate ═ 2.01 [95% CI 1.86–2.18], *P* < 0.001) and low (rate ratio estimate ═ 2.25 [95% CI 2.07–2.45], *P* < 0.001) risk groups. This analysis suggests that the PRODIGY score may be useful in assessing the risk of PACU RDEs. Trial Registration: https://www.clinicaltrials.gov/ct2/show/NCT02707003.

## Introduction

The initial phase of anesthesia recovery is complex, where vital organ systems regain normal function from the effects of general anesthesia and surgery. Typically, this process occurs in the postanesthesia care unit (PACU), a specialized unit where patients receive intensive care from specialized nurses under the supervision of an anesthesiologist [1]. Emerging evidence has found strong correlations between respiratory depression episodes (RDEs), such as episodes of apnea, occurring during the PACU admission, and the subsequent development of potentially life-threatening episodes of respiratory failure on general care postoperative wards [2]. Preoperative assessment of patient risk for PACU RDEs could be of clinical benefit to the anesthesiologist in developing an anesthetic plan to mitigate this risk; however, no such scoring tools have been assessed.

The PRediction of Opioid-induced respiratory Depression In patients monitored by capnoGraphY (PRODIGY) trial was a large, prospective multinational trial that utilized continuous capnography and pulse oximetry to monitor patients in general care wards to develop a prediction model for RDE risk [3]. In that study, RDE was defined as respiratory rate ≤ 5 breaths/min, oxyhemoglobin saturation ≤ 85%, or end-tidal carbon dioxide ≤ 15 or ≥ 60 mm Hg for ≥ 3 min, apnea episode > 30 s, or any respiratory opioid-related adverse episode, such as opioid reversal, respiratory failure, or cardiopulmonary arrest. Using these data, a multivariable respiratory depression prediction model (PRODIGY score) was developed using five independent variables (age, sex, history of sleep-disordered breathing, chronic heart failure, and opioid naïvety [Table S1]). The PRODIGY score can be used to categorize patients as low, intermediate, or high risk for RDEs in the general care wards [3].

Previously, Chung et al. [4] utilized continuous capnography and pulse oximetry on adult postsurgical patients to characterize RDEs in the PACU, following general anesthesia. Because this dataset utilized the same technology (capnography and pulse oximetry) to diagnose RDEs as used in the PRODIGY trial, it provides a unique opportunity to assess if the PRODIGY score can be used to assess risk for PACU RDEs. In the current study, we conducted a post-hoc analysis of a subset of the subjects enrolled in [4] to explore if there is a rationale to consider whether the PRODIGY score is associated with RDEs in the PACU.

**Table 1:** Types of respiratory adverse episodes and frequency of respiratory episodes in PACU

**Monitored respiratory adverse episode**	**Level II episode**	**Patients with episode, *n* (%)**	**Level II episodes, *n***	**Level II episodes, median [IQR]**	**Level I episode**	**Patients with episode, *n* (%)**	**Level I episodes, *n***	**Level I episodes, median [IQR]**
Tachypnea	≥25 bpm >15 s	33 (38)	244	2 [1,10]	≥30 bpm >30 s	3 (3)	12	3 [1,8]
Bradypnea	≤ 8 bpm >15 s	50 (57)	468	8 [2,15]	≤6 bpm >30 s	25 (29)	183	5 [2,11]
Hypercapnia	≥55 mmHg >15 s	10 (11)	121	5 [3,16]	≥60 mmHg >30 s	1 (1)	2	2 [2,2]
Hypocapnia	≤25 mmHg >15 s	29 (33)	248	7 [2,12]	≤25 mmHg >30 s	22 (25)	184	7 [4,13]
Tachycardia	≥120/min for 15 s	5 (6)	23	3 [1,9]	≥120/min >30 s	7 (8)	19	1 [1,2]
Bradycardia	≤40/min for 15 s	29 (33)	33	1 [1,1]	≤40/min >30 s	0 (0)	0	0 [0,0]
Hypoxemia	≤90% >15 s	38 (44)	224	3 [1,7]	≤90% >30 s	27 (31)	130	2 [1,5]
Apnea	≥10 s per 15 min	74 (85)	1689	13 [4,28]	≥10 s twice per 15 min	0 (0)	0	0 [0,0]

Level I and Level II alerts are categories of RDEs, with Level I defined as requiring immediate physician attention and Level II defined as requiring nursing attention. bpm: Breaths per minute; IQR: Interquartile range; RDEs: Respiratory depressive episodes; PACU: Postanesthesia care unit.

## Materials and methods

We performed a post-hoc analysis of the Chung et al. [4] dataset to assess whether the PRODIGY score is associated with RDEs in the PACU.

### Participants

The original trial was designed to include up to 250 patients across two sites. This post-hoc analysis was performed using patient data from the United States trial site only, including the 87 patients who completed the trial and had complete (≥90%) monitoring data at that site [4]. As in the original trial, patients with more than 10% of continuous monitoring data missing were excluded from the analysis cohort, as imputation of the missing monitoring data was not feasible. Data from the second trial site was not available for post-hoc evaluation due to data privacy restrictions.

### Trial design and objective

The trial was designed to quantify the occurrence of respiratory episodes in patients in the PACU. As previously described [4], the inclusion criteria were as follows: (1) adults ≥18 years; (2) an American Society of Anesthesiologists (ASA) score of II–IV; (3) patients scheduled for elective surgery requiring general anesthesia; (4) duration of general anesthesia for >1.5 h; (5) requirement of intraoperative opioids; (6) PACU stay of ≥ 45 min; and (7) expected transfer from the PACU to an inpatient setting. The exclusion criteria were as follows: (1) ambulatory procedures; (2) physical inability to wear oral/nasal capnography sampling filter line or finger sensors; or (3) pregnancy.

The anesthetic management was under the direction of the attending anesthesiologist and included maintenance with a volatile anesthetic and analgesia with fentanyl. The neuromuscular blockade, if indicated, was reversed at the conclusion of the procedure, and midazolam was administered preoperatively if needed for anxiolysis. After being transferred to the PACU, patients were monitored using standard care monitoring equipment, along with blinded capnography and pulse oximetry (Capnostream™ 20p monitor, Nellcor™ Max A pulse oximeter, and Microstream™ Smart CapnoLine™ Plus O2 sampling line [Medtronic, Inc., Boulder, CO, USA]). All enrolled patients were required to undergo capnography monitoring for at least 45 min. Monitoring parameters were predefined to detect RDEs, triggering either Level I alerts (requiring immediate physician attention, such as administering naloxone or applying noninvasive positive pressure ventilation) or Level II alerts (requiring nursing attention, such as increasing supplemental oxygen or repositioning the patient). Specific definitions of Level I and Level II alerts are provided in Table 1 [4].

The five clinical variables required to calculate the PRODIGY score (age, sex, history of heart failure, history of obstructive sleep apnea [OSA], and opioid naïvety) were available for all subjects. Each variable is assigned points which are summated to calculate the PRODIGY score (Table S1) with a score of <8 being low risk, ≥8 and <15 intermediate risk, and ≥15 high risk for RDE in general care wards [3].

**Figure 1.**
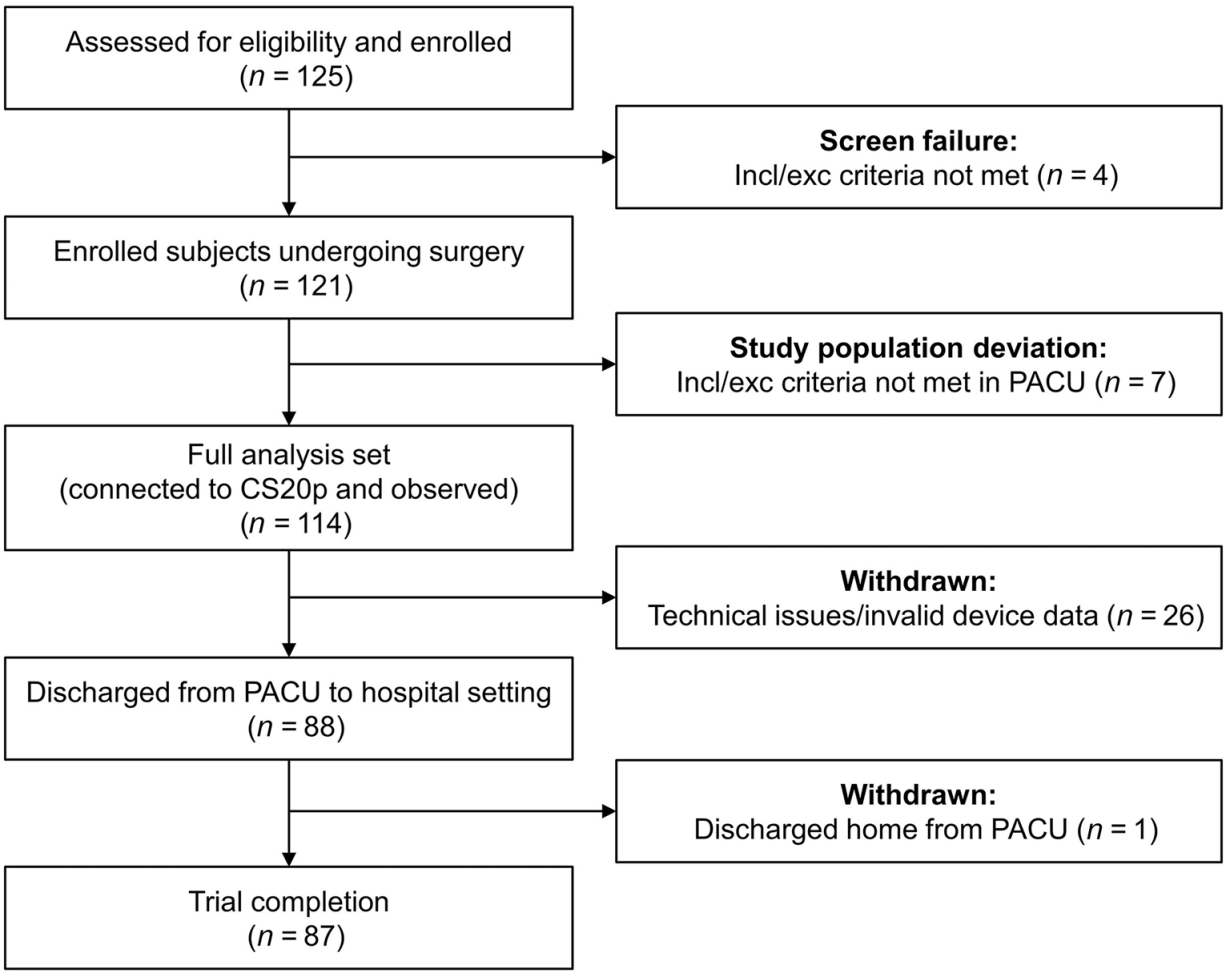
**Flowchart of patients enrolled at one United States trial site.** PACU: Post anesthesia care unit; Incl: Inclusion; Exc: Exclusion; CS20p: Capnostream™ 20p monitor.

### Outcomes measures

The objective of the original PACU trial was to quantify the occurrence of respiratory episodes, including apnea, hypoxemia, hypercapnia, hypocapnia, tachypnea, bradypnea, tachycardia, and bradycardia in surgical patients. Each anticipated respiratory episode was defined a priori, including alert thresholds appropriate for nurses (Level II) and physicians (Level I) [4]. The objective of this post-hoc analysis was to assess whether the PRODIGY score is associated with the incidence of respiratory episodes in the PACU.

### Ethical statement

This study was conducted in accordance with the Declaration of Helsinki and all local regulatory requirements. The protocol was approved by the institutional review board of all participating sites (Toronto Western Hospital and University of Arizona Medical Center). The original trial was registered with www.clinicaltrials.gov under NCT02707003 (Principal Investigators: Peter R. Lichtenthal, Frances F. Chung; Registered March 11, 2016). All subjects provided written informed consent. This manuscript adheres to the Strengthening the Reporting of Observational Studies in Epidemiology (STROBE) guidelines.

### Statistical analysis

Analyses were performed using SAS^®^ Version 9.4 (SAS Institute Inc., Cary, NC, USA) and data were summarized by descriptive statistics (mean with standard deviation or median with interquartile range [IQR] for continuous variables) and frequencies and percentages for categorical variables. The analysis of variance (ANOVA) and Chi-Square tests were used to compare the baseline characteristics across PRODIGY groups. Because the outcome of interest, PACU RDEs, was skewed, analysis across PRODIGY groups was performed with the Kruskal–Wallis test. To account for varying lengths of PACU stay, Poisson regression was used to assess whether the rate of RDEs differed between PRODIGY groups. For this analysis, the RDE count was the dependent variable, the PRODIGY group was the independent variable, and the log of PACU stay duration was included as an offset to account for patients having different PACU stay durations. Results from this analysis are summarized by presenting the rate ratio estimate with a 95% confidence interval for the pairwise PRODIGY group comparisons.

## Results

### Participants

Of the 125 patients assessed for eligibility and enrolled at a single site in the United States, 87 patients completed the trial (Figure 1). Eleven patients did not meet the trial inclusion and exclusion criteria, and 26 were withdrawn due to technical issues (study coordinator or monitoring equipment unavailable at the time of PACU admission) or invalid device data (less than 90% of continuous monitoring data). All 87 patients with valid device data were included in this post-hoc analysis. Patient and procedural characteristics are shown in Table 2. Even though the trial was open to ASA IV patients, none were recruited from the center included in this subset.

**Table 2:** Patient clinical and demographic characteristics

	**PRODIGY risk group**				
**Characteristic**	**Overall, *n* ═ 87**	**Low, *n* ═ 26 (30)**	**Intermediate, *n* ═ 29 (33)**	**High, *n* ═ 32 (37)**	***P***
Age (years)	57 ± 14	45 ± 12	54 ± 12	69 ± 5	<0.001
Sex					<0.001
Male	34 (39)	0	13 (45)	21 (66)	
Female	53 (61)	26 (100)	16 (55)	11 (34)	
Body mass index (kg/m^2^)	29 ± 7	31 ± 9	28 ± 6	28 ± 7	0.21
ASA Score*					0.07
II	44 (51)	16 (62)	17 (59)	11 (34)	
III	43 (49)	10 (38)	12 (41)	21 (66)	
Medical history					
Cardiovascular	61 (70)	11 (42)	19 (66)	31 (97)	<0.001
Respiratory**	38 (44)	10 (38)	13 (45)	15 (47)	0.80
Obstructive sleep apnea	15 (17)	1 (4)	6 (21)	8 (25)	0.09
Non-invasive pressure at night	7 (8)	0	2 (7)	5 (16)	
Surgery type					0.03
Orthopedic/plastics	38 (44)	6 (23)	17 (59)	15 (47)	
General	18 (21)	4 (15)	8 (28)	6 (19)	
Urology/gynecology	15 (17)	9 (35)	3 (10)	3 (9)	
Otorhinolaryngology	8 (9)	4 (15)	1 (3)	3 (9)	
Thoracic	5 (6)	1 (4)		4 (13)	
Craniotomy	3 (3)	2 (8)		1 (3)	
Anesthesia duration (min)	235 ± 131	240 ± 119	272 ± 155	196 ± 109	0.08
Length of stay in PACU (min)	166 ± 92	178 ± 94	162 ± 114	160 ± 69	0.75
Intensive care unit admission	15 (17)	2 (8)	2 (7)	11 (34)	0.006
Transport from PACU on oxygen	63 (72)	14 (54)	22 (76)	27 (84)	0.03

Data are presented as mean ± standard deviation for continuous variables and as number (percentage) for categorical variables. ^*^No ASA IV patients were enrolled from this medical center; ^**^Respiratory disorders included OSA, chronic obstructive pulmonary disorder, asthma, or other pulmonary/respiratory diseases. ASA: American Society of Anesthesiologists; PACU: Postanesthesia care unit; PRODIGY: PRediction of Opioid-induced respiratory Depression In patients monitored by capnoGraphY; SD: Standard deviation; OSA: Obstructive sleep apnea.

### Respiratory episodes in the PACU

During anesthesia recovery, 3580 respiratory episodes occurred during blinded capnography monitoring, including 3050 Level II alerts and 530 Level I alerts. The most common alert was the Level II apnea alert, occurring in 85% of patients (*n* ═ 74/87), with a median (IQR) of 13 [4–28] alerts during the PACU stay (Table 1). Bradypnea, hypocapnia, tachypnea, and hypoxemia Level II alerts were also common. Hypoxemia and bradypnea were the most common Level I alerts (Table 1).

### Association of PRODIGY score and respiratory episodes

**Figure 2.**
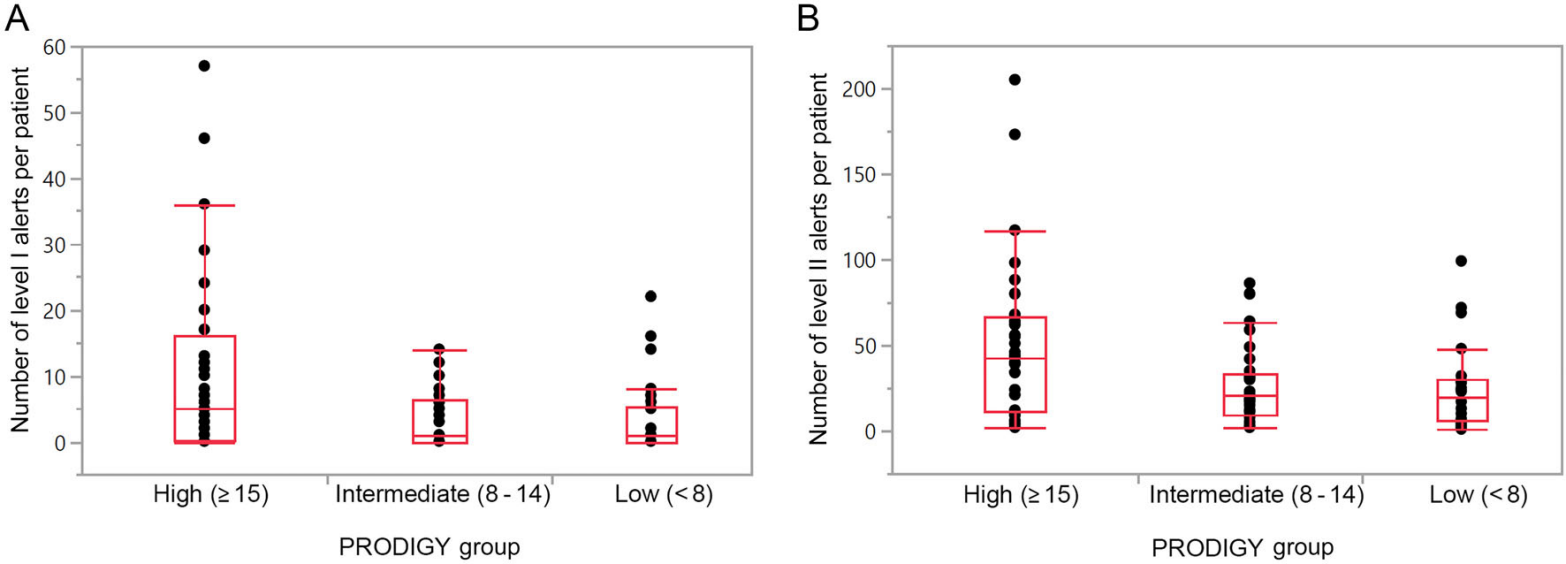
**Assessment of Level I (A) and Level II (B) alerts by PRODIGY risk group (high, intermediate, or low).** Level I and Level II alerts are categories of respiratory depression, with Level I defined as requiring immediate physician attention and Level II defined as requiring nursing attention. PRODIGY: PRediction of Opioid-induced respiratory Depression In patients monitored by capnoGraphY.

Twenty-six (29.9%) patients had low, 29 (33.3%) intermediate, and 32 (36.8%) high PRODIGY scores (Table 3). The total number and duration of alerts were highest in the high PRODIGY risk group patients (*P* ═ 0.035 and *P* ═ 0.042, respectively) (Table 3). Among patients who had a Level I alert, those in the high PRODIGY risk group had significantly more episodes (*P* ═ 0.046) (Figure 2A). Level II alerts were also most common in patients in the high PRODIGY risk group (*P* ═ 0.057) (Figure 2B). From Poisson regression, the rate of RDE for patients in the high PRODIGY risk group was significantly higher than that for patients in the low (rate ratio ═ 2.25, 95% CI 2.07–2.45; *P* < 0.001) and intermediate (rate ratio ═ 2.01, 95% CI 1.86–2.18; *P* < 0.001) risk groups, and the rate of RDE in patients in the intermediate-risk group was significantly higher than in patients within the low-risk group (rate ratio ═ 1.12, 95% CI 1.02–1.23; *P* ═ 0.022).

**Table 3:** PRODIGY risk group and number of RDE alerts detected during anesthesia recovery

**Alert type**	**PRODIGY risk group**			
	**Low, *n* ═ 26**	**Intermediate, *n* ═ 29**	**High, *n* ═ 32**	***P***
*Total alerts*				
Patients with alert	26 (100)	29 (100)	32 (100)	–
Alerts per patient	22 [9,37]	26 [13,42]	56 [12,87]	0.035
Duration, s	158 [60,554]	420 [143,761]	712 [139,1456]	0.042
Alerts per hour	8.8 [3.2,15.4]	10.9 [3.7,18.2]	21.5 [5.6,37.1]	0.027
*Level I alerts*				
Patients with alert	15 (58)	17 (59)	24 (75)	0.287
Alerts per patient*	5 [1,8]	5 [2,8]	9 [4,20]	0.046
Duration, s^*^	177 [30,779]	301 [168,702.5]	303 [123,885]	0.356
Alerts per hour	1.6 [0.4,5.1]	2.3 [0.8,4.5]	4.5 [1.3,8.2]	0.055
*Level II alerts*				
Patients with alert	26 (100)	29 (100)	32 (100)	–
Alerts per patient	20 [7,30]	21 [10,34]	43 [11,66]	0.057
Duration, s	102 [53,302]	199 [55,484]	401 [76,733]	0.054
Alerts per hour	7.3 [3.1,14.6]	9.4 [3.4,16.5]	16.8 [5.0,29.9]	0.053

Level I and Level II alerts are categories of respiratory depression, with Level I defined as requiring immediate physician attention and Level II defined as requiring nursing attention. Values are presented as *n* (%), or median [IQR]. ^*^Values are calculated only from patients who experienced ≥ 1 Level I alert. IQR: Interquartile range; PRODIGY: Prediction of Opioid-Induced Respiratory Depression in Patients Monitored by Capnography; RDEs: Respiratory depressive episodes.

## Discussion

This study found that the number of RDEs in the PACU increases with higher PRODIGY scores. Importantly, the rate of Level I episodes, the more serious episodes that would prompt physician intervention, was significantly greater in patients in the high PRODIGY group. These findings suggest that the PRODIGY score may have utility in assessing the risk of postoperative respiratory depression (PRD) during anesthesia recovery. Our results are similar to a post-hoc analysis of nursing-diagnosed RDEs in the PACU following laparoscopic surgery, where high vs low PRODIGY groups were associated with an increased risk of PRD (odds ratio ═ 1.46) [5].

Failure to identify and treat PRD can lead to cardiopulmonary arrest, anoxic brain injury, and death [6, 7]. While continuous monitoring of all postoperative patients has been shown to improve patient outcomes, this may not be feasible in all practices or in low-resource/low-staff settings [8, 9]. Surgical patients who have a positive assessment screen for OSA and experience respiratory depression in the PACU have higher rates of postoperative pulmonary complications compared to other surgical patients [10]. Evaluation of PRODIGY scores to assess the risk of PRD, perhaps in conjunction with the Snoring, Tiredness, Observed apnea, high blood Pressure, Body mass index, Age, Neck circumference, and Gender (STOP-BANG) scores [11] to assess OSA, in the PACU can guide postoperative monitoring decisions and help optimize the perioperative management of surgical patients based on individual needs. This could include continuous monitoring in the PACU and surgical unit or, in some cases, may contribute to the decision to transfer a high-risk patient to a higher acuity unit.

Other studies have found that patients with respiratory depression in the PACU have a five-fold increased risk of requiring naloxone to treat severe opioid toxicity in the general care ward [1, 12]. Continuous measurements of minute volume found that patients with depressed ventilation in the PACU continued to have depressed ventilation for hours once discharged to general care wards [13]. This relationship between RDEs in the PACU and the ward suggests that the PRODIGY score may have utility in identifying patients who are at increased risk of developing respiratory depression in the PACU. Postoperative patients in the high PRODIGY group should undergo closer nursing observation in the PACU for signs of respiratory depression, and those patients who experience an RDE in the PACU should be carefully evaluated prior to discharge to general care wards to determine if enhanced postoperative monitoring and care are indicated.

Based on prior studies, enhanced monitoring may be most beneficial on the first postoperative day. Recent studies have found that RDEs begin to occur shortly after PACU discharge to general care wards [13, 14]. Studies examining the use of postoperative naloxone administration to reverse severe opioid toxicity found that most naloxone was given in the afternoon and evening following surgery [2].

This study has several limitations. Firstly, this was a subanalysis of a previous pilot study, as data from only one site were available for analysis. This limitation reduced the statistical power to determine if the PRODIGY score would correlate with postoperative outcomes [4]. Given the limited sample size and single setting of the current study, additional studies should be performed to confirm the generalizability of these findings. Secondly, capnography and pulse oximetry monitoring were confined to the PACU only, so we can only speculate that patients who had respiratory depression in the PACU continued to experience it on the wards. Thirdly, ASA I and IV patients were not included in this analysis. Inclusion or subset analysis of ASA IV patients may further reveal risk factors that lead to an increased risk of RDEs. Lastly, because the anesthetic care was left to the discretion of the attending anesthesiologist, we cannot account for whether perioperative management was altered based on the perception of risk for PACU RDEs.

## Conclusion

A high PRODIGY score, designed to detect RDEs in general care wards, may be associated with RDE occurrence in the PACU. These observations suggest the potential utility of the PRODIGY score in assessing the risk for early RDEs occurring during anesthesia recovery, but further study is required to determine if these findings can be generalized to other populations.

## Prior presentation

A portion of this content was selected and presented as a top abstract at the 2022 Society of Anesthesia and Sleep Medicine annual meeting (Virtual, October 21, 2022).

## Supplemental data

**Table S1:** PRODIGY risk group calculation

**Patient characteristic**	**Points assigned**	**Example patient 1**	**Example patient 2**
Age 60–69	8		X
Age 70–79	12		
Age >79	16		
Male sex	8	X	
Sleep disordered breathing	5	X	
Opioid naïve	3		X
Chronic heart failure	7	X	
*Sum*	PRODIGY score	20 (high risk)	11 (intermediate risk)

A PRODIGY score of less than 8 points is considered low risk, 8–14 points is considered intermediate risk, and more than 14 points is considered high risk for respiratory depression episodes. PRODIGY: PRediction of Opioid-induced respiratory Depression In patients monitored by capnoGraphY.

## Data Availability

All data generated or analyzed during this study are included in this published article and its supplementary information files.
